# Discovery of Novel Transketolase Epitopes and the Development of IgG-Based Tuberculosis Serodiagnostics

**DOI:** 10.1128/spectrum.03377-22

**Published:** 2023-01-18

**Authors:** Jaya Talreja, Changya Peng, Tuan-Minh Nguyen, Sorin Draghici, Lobelia Samavati

**Affiliations:** a Department of Medicine, Division of Pulmonary, Critical Care and Sleep Medicine, Wayne State University School of Medicine and Detroit Medical Center, Detroit, Michigan, USA; b Department of Computer Science, Wayne State University, Detroit, Michigan, USA; c Center for Molecular Medicine and Genetics, Wayne State University School of Medicine, Detroit, Michigan, USA; University of Brescia

**Keywords:** tuberculosis, *Mycobacterium tuberculosis*, sarcoidosis, IgG, transketolase epitopes, peptide ELISA

## Abstract

Despite advances in rapid molecular techniques for tuberculosis (TB) diagnostics, there is an unmet need for a point-of-care, nonsputum-based test. Previously, through a T7 phage antigen display platform and immunoscreening, we identified that the serum IgGs of active TB patients differentially bind to several antigen-clones and that this immunoreactivity discriminates TB from other respiratory diseases. One of these high-performance clones has some homology to the transketolase of Mycobacterium tuberculosis (*M.tb* TKT). In this study, we developed a direct enzyme-linked immunosorbent assay (ELISA) detecting IgG against the TKT antigen-clone (TKTμ). Through sequence alignment and *in silico* analysis, we designed two more peptides with potential antigenicity that correspond to *M.tb-*specific transketolase (*M.tb* TKT1 and *M.tb* TKT3) epitopes. After the development and standardization of a direct peptide ELISA for three peptides, we tested 292 subjects, including TB (*n* = 101), latent tuberculosis infection (LTBI) (*n* = 49), healthy controls (*n* = 66), and sarcoidosis (*n* = 76). We randomly assigned 60% of the subjects to a training set to create optimal models to distinguish positive TB samples, and the remaining 40% were used to validate the diagnostic power of the IgG-based assays that were developed in the training set. Antibodies against *M.tb* TKT3 yielded the highest sensitivity (0.845), and these were followed by TKTμ (0.817) and *M.tb* TKT1 (0.732). The specificities obtained by TKTμ, *M.tb* TKT3, and *M.tb* TKT1 on the test sets were 1, 0.95, and 0.875, respectively. The model using TKTμ obtained a perfect positive predictive value (PPV) of 1, and this was followed by *M.tb* TKT3 (0.968) and *M.tb* TKT1 (0.912). These results show that IgG antibodies against transketolase can discriminate active TB against LTBI, sarcoidosis, and controls.

**IMPORTANCE** There is an unmet need for a point-of-care, nonsputum-based TB test. Through the immunoscreening of a novel T7 phage library, we identified classifiers that specifically bind to IgGs in active TB sera. We discovered that one of these clones is aligned with Mycobacterium tuberculosis transketolase (TKT). TKT is an essential enzyme for Mycobacterium tuberculosis growth. We designed three TKT epitopes (TKTμ, TKT1, and TKT3) to detect TKT-specific IgGs. After the development and standardization of three different ELISA-utilizing TKT peptides, we tested 292 subjects, including active TB, LTBI, healthy controls, and sarcoidosis. Rigorous statistical analyses using training and validation sets showed that ELISA-based detections of specific IgGs against TKT3 and TKTμ have the greatest sensitivity, specificity, and accuracy to distinguish active TB subjects from others, even LTBI. Our work provides a novel scientific platform from which to further develop a point-of-care test, thereby aiding in faster TB diagnoses.

## INTRODUCTION

Active *Mycobacterial tuberculosis* infection (TB) remains a global health threat, with 10 million new cases and 1.7 million deaths annually (1, 2). One-third of the world’s population is infected with TB, but not all are considered to have latent TB infection (LTBI) (2). Pulmonary TB is contagious and can be lethal, and LTBI can evolve into active TB (1). Efforts during the past decade to consistently diagnose and treat pulmonary cases have slowed the TB incidence rate, but they have not yielded substantial progress (3). The existing TB diagnostics pipeline still does not have a simple, rapid, inexpensive, point-of-care (POC) test (3). The World Health Organization (WHO) has defined high-priority target product profiles for tuberculosis diagnostics (4, 5). These priorities include a sputum-based POC smear replacement test, a nonsputum biomarker-based POC TB test, a POC triage test, and a rapid drug-susceptibility test (DST) (5). The discoveries of specific biomarkers in the form of antibodies distinguishing the immunity in active TB and LTBI could be the keys to understanding the humoral responses against mycobacterial pathogens. Current commercially available antibody-based TB tests show poor sensitivity and specificity, and none can distinguish active TB from LTBI (6). Due to the lack of precision, the WHO does not endorse the routine application of the current commercial serological tests for TB diagnosis (7, 8). The serological diagnosis of tuberculosis has been challenging, partly due to the large *M.tb* proteome, the even larger antigenic epitopes (4), and the heterogeneity across humans in their immunoglobulin G (IgG) responses to tuberculosis (9). Most tuberculosis serological tests use purified *M.tb* proteins and a single or small number of antigens that are predominantly derived from the membrane proteins of *M.tb* (6). The challenges for the development of effective serological tests include the need to differentiate active pulmonary TB from other pulmonary diseases, including pneumonia or other granulomatous diseases, such as pulmonary sarcoidosis.

Sarcoidosis is a non-infectious systemic granulomatous disease with remarkable similarity to TB in clinical, immune responses and in gene expression signatures (10–13). Previously, we developed a T7 phage antigen display platform, and after the immunoscreening of large sets of serum samples, we identified 10 clones that differentially bind to the serum IgG of active TB patients, thereby differentiating TB from other respiratory diseases (14, 15). One of these high-performance clones had homology to the transketolase of Mycobacterium tuberculosis (*M.tb*) (14, 16). Transketolase (TKT) is an essential enzyme for the intracellular growth of *M.tb* (17), and it is the key enzyme in the non-oxidative pentose phosphate pathway (17–19). It catalyzes the reversible transfer of a two-carbon ketol group from sedoheptulose-7-phosphate to glyceraldehyde-3-phosphate, thereby producing xylulose-5-phosphate and ribose-5-phosphate. TKT has a highly evolutionarily conserved structure and function, but there are intraspecies and interspecies amino acid (AA) variations among microorganisms and mammals (17, 19). Because we have previously shown that the IgG of TB sera differentially bind to an antigen-clone containing TKTμ (14, 15), and because the transketolase of *M.tb* is required for bacterial growth (20), we hypothesize that an abundance of IgG in sera against specific TKT may differentiate between active TB, LTBI, and other non-TB granulomatous lung diseases. By aligning TKTμ with TKT sequences from H. sapiens, *M.tb*, and Staphylococcus aureus, we found that the core sequence of TKTμ shares homology with *M.tb* TKT. Because there are some AA differences between TKTμ and *M.tb* TKT, we designed two additional *M.tb* TKT-peptide homologs (TKT1 and TKT3) to develop a novel direct ELISA and to quantify the levels of IgG against these three TKT peptides (TKTμ, *M.tb* TKT1, and *M.tb* TKT3) for the serodiagnosis of TB.

## RESULTS

### Selection of a novel tuberculosis antigen and development of ELISA.

Through the immunoscreening of our microarray platform with two different sets of sera from active TB and various positive and negative controls, we identified 10 highly performing clones (14, 15). One of those clones had homology to *M.tb* TKT, and we labeled that sequence as TKTμ. Because of the high sensitivity of TKTμ to recognize the IgG of TB sera, we chose this clone to develop a rapid ELISA for the serodiagnosis of TB (14, 15). To develop the ELISA, we chemically synthesized and purified the TKTμ peptide. After extensive optimizations of peptide concentration, plate selection, serum dilution, incubation parameters, and detection antibody, we determined the avidity of specific IgGs binding against the TKTμ epitope in the sera of study subjects (*n* = 292) via direct ELISA. The avidity is the functional affinity as an indicator of the relative strength of the interactions between IgG antibody binding sites to the TKTμ epitope. In addition to healthy controls and TB patients, we included LTBI subjects and patients with sarcoidosis. We included sarcoidosis subjects because many studies suggested a shared underlying pathophysiology and clinical features between sarcoidosis and TB (10, 11, 13). For instance, *M.tb* antigens (ESAT6 and MKatG) induce T-cell-specific responses in the blood of sarcoidosis patients (12, 21, 22). All of the TB subjects had positive AFB smear and culture tests, whereas LTBI was defined based on a negative smear and a negative culture but a positive interferon gamma release assay (IRG) and positive skin tests. The characteristics of the study groups are shown in Table 1.

**TABLE 1:** Study subjects

Characteristic	Controls	Sarcoidosis	TB subjects	LTBI
Age (Mean ± SEM^*a*^)	41 ± 11	46 ± 12	35 ± 11	33 ± 10
Gender, N (%)	66	76	101	49
Male	27 (40)	27 (35)	67 (66)	30 (61)
Female	39 (60)	49 (64)	34 (34)	19 (39)
Race, N (%)				
African-American	21 (31)	72 (95)	0	0
African	4 (6)	0	11 (10)	9 (18)
White	29 (43)	3 (4)	0	0
Asians	13 (19)	1 (1)	89 (90)	40 (82)
IRGs^*c*^	NA^*b*^	negative	NA	positive
TB smear^*d*^	NA	N/A	positive	negative

a Standard error of mean.

b Not applicable.

c Interferon γ release assay.

d TB Smear (AFB positive sputum).

**FIG 1:**
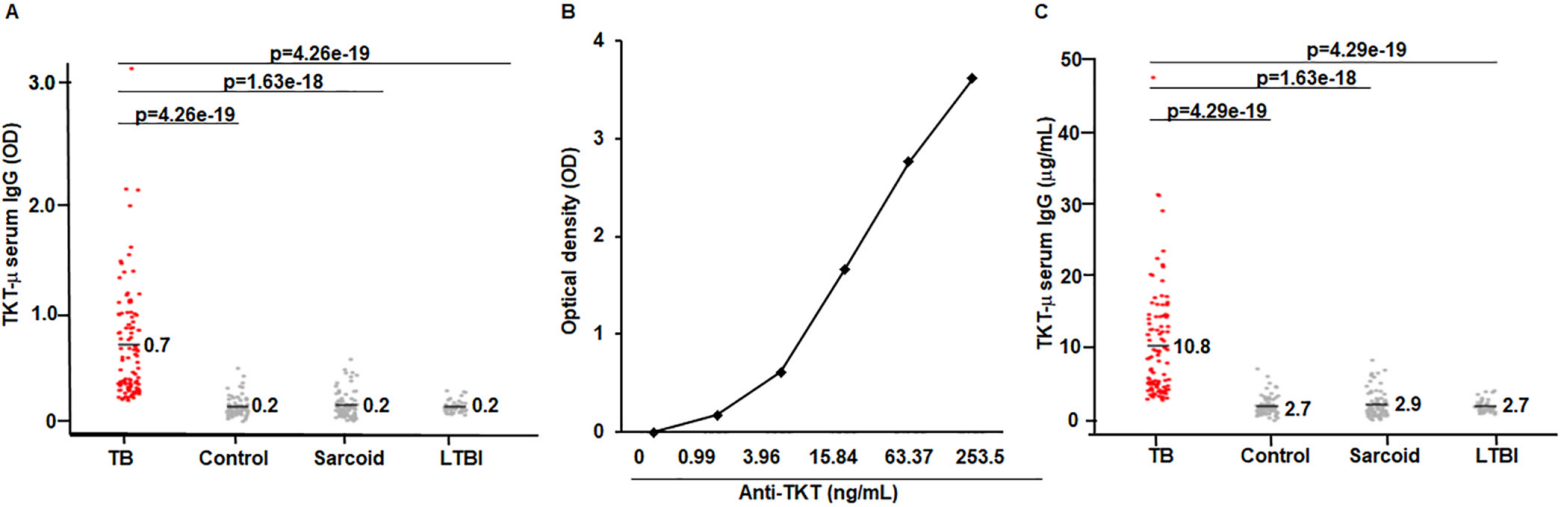
Detection and quantification of TKTμ-specific IgGs. (A) TKTμ-specific IgGs were measured via standardized, direct TKT peptide ELISA. The OD value at 450 nm was a measure of the TKTμ-specific IgGs in the sera from all study groups, namely, healthy controls (*n* = 66), sarcoid (*n* = 76), smear positive TB subjects (*n* = 101), and LTBI (*n* = 49), via ELISA. The TB patients showed significantly higher OD values, compared to the healthy controls (*P* = 4.26E−19), sarcoidosis (*P* = 1.63E−18), and LTBI (*P* = 4.26E−19). (B) Polyclonal anti-TKTμ IgGs were generated by immunizing rabbits with TKTμ peptide, and they were used to obtain a standard curve for TKTμ peptide ELISA. The anti-TKTμ IgG standard curve showing the OD at 450 nm versus the anti-TKTμ IgG concentration (ng/mL). (C) The anti-TKTμ IgG concentration (ng/mL) in all study groups; TB patients displayed significantly higher levels of serum TKTμ IgGs (10.8 ± 7.25) compared to control (2.7 ± 1.32), sarcoid (2.9 ± 1.77), and LTBI (2.7 ± 0.821). After the correction of *P* values for multiple comparisons, the TKTμ IgGs levels in TB sera were significantly different, compared to the control (*P* = 1.63E−18), sarcoid (*P* = 1.63E−18), or LTBI (*P* = 4.26E−19).

All measurements were duplicates and were standardized in our lab. The results presented in Fig. 1A show the serum IgG optical density (OD) values against the TKTμ epitope among study subjects. The OD values (mean ± SD) of the TKTμ-specific IgGs in the sera of the TB patients was 0.7 ± 0.48, whereas the OD values in healthy controls, sarcoidosis, and LTBI had average OD values of 0.18 ± 0.09, 0.19 ± 0.12, and 0.18 ± 0.05, respectively. As shown, there were significant differences in the OD values between TB and the healthy controls (adjusted [adj.] *P* = 4.26E−19), sarcoidosis (adj. *P* = 1.63E−18) and LTBI (adj. *P* = 4.26E−19).

### Rabbit immunizations and polyclonal antibodies against TKTμ.

Because we observed significant differences in the IgG immunoreactivity of the TB sera against the TKTμ epitope, we immunized rabbits with the TKTμ peptide to quantify the concentration of the TKTμ-specific IgGs in their sera and to develop a TKTμ standard curve. After 3× immunizations of rabbits with the TKTμ peptide, the immunization achieved a high concentration of polyclonal IgG antibodies against TKTμ that was purified. The polyclonal IgG antibody against TKTμ (anti-TKTμ IgG) was then utilized to obtain an anti-TKTμ antibody standard curve (Fig. 1B). The results of the TKT peptide ELISA using different dilutions (1:1000 to 1:512,000) of rabbit anti-TKTμ IgG are shown in Table S1. In the study groups, the concentration of TKTμ specific IgGs were calculated based on standard curves. The mean of the anti-TKTμ IgG antibodies in the TB sera was 10.8 (ranging from 3 μg to 50 μg/mL). The mean of the anti-TKTμ IgG antibodies in the sera of the controls, LTBI, and sarcoidosis patients was 2.7 (ranging from 1.7 to 3.5 μg/mL). The false discovery rate (FDR)-adjusted *P* values were: between the TB samples and the healthy control values, *P =* 4.29E−19, between the TB samples and sarcoidosis, *P* = 1.6E−18; between the TB samples and LTBI, *P* = 4.29E−19. These data suggest that a significant difference in the level of anti-TKTμ IgG antibody values in the sera of the TB subjects exists, even compared to the LTBI values (Fig. 1C).

### TKT-sequence alignments of Mycobacterium tuberculosis, Homo sapiens, *and* Staphylococcus aureus.

**FIG 2:**
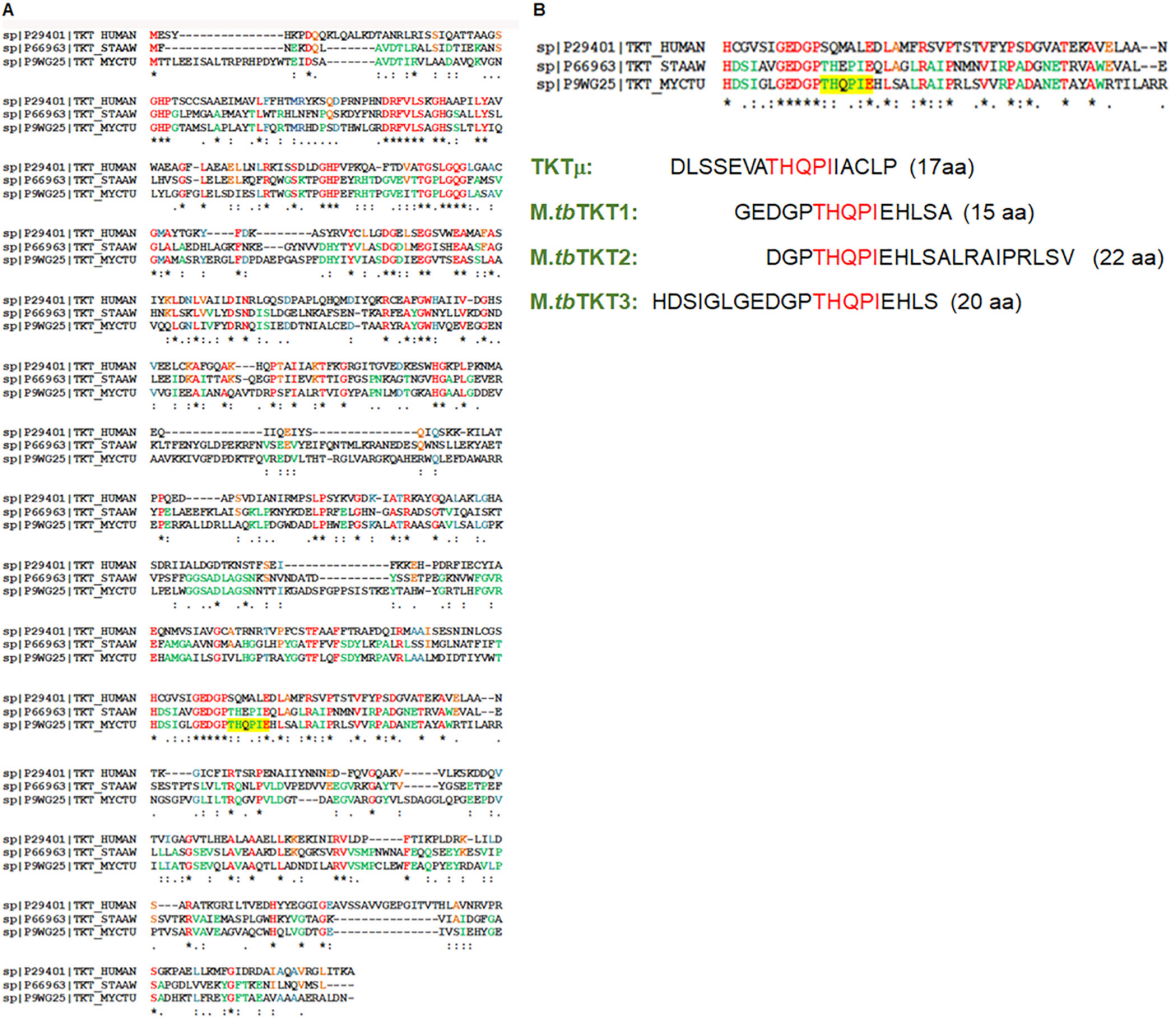
Sequence alignment of TKT from Mycobacterium tuberculosis, Staphylococcus aureus, and Homo sapiens. The amino acid sequences of TKT for all three species were obtained from UniProtKB: P9WG25 (TKT_MYCTU), A0A0B4J1R6 (TKT_HUMAN), and Q6G9L6 (TKT_STAAS). The sequence alignment and homology of TKT was done using the T-Coffee Multiple Sequence Alignment program. (A) CLUSTALW (1.83) multiple sequence alignment. Identical residues in all three species are indicated in red, identical residues for *M.tb* and in green for S. aureus. (B) Sequence of TKTμ and designed peptides, *M.tb* TKT1, *M.tb* TKT2, and *M.tb* TKT3, corresponding to *M.tb* TKT epitopes. The discovered TKTμ peptide (17 AA) shows a sequence similarity of THQPI AAs with *M.tb* TKT.

TKT is an evolutionarily conserved enzyme that is ubiquitously present from microorganisms to humans, but there are interspecies and intraspecies variations in gene and protein sequences. Therefore, we aligned TKTμ with the TKT of *M.tb*, Homo sapiens, and Staphylococcus aureus (Fig. 2). The TKT structure from most species is arranged into three domains: Domains I (1 to 322), II (323 to 527), and III (528 to 700). Domain III comprises the last approximately 170 amino acids and is involved in the regulation of the enzyme activity, and most mutations have been reported in this domain (17). *M.tb* TKT has 26% sequence homology with Homo sapiens and 43.8% sequence homology with S. aureus transketolase. We found that our discovered TKTμ peptide (17 AA) has similarity to the *M.tb* transketolase on the AA sequence of THQPI, spanning from AAs 562 to 566 of *M.tb* TKT (Fig. 2B). It is the core domain III of *M.tb* TKT, which is the part of the open reading frame that contains the specific sequence motif of THDSIGLGEDGPTHQPIE [17]. Because of the detection of the abundance of the IgG antibody against the TKTμ epitope in the sera of TB patients, we investigated whether designing peptides corresponding only to *M.tb* TKT, but not the other organisms, could improve the sensitivity and specificity of the ELISA. Therefore, we designed three other peptides containing epitopes spanning the AAs 541 to 570 of the *M.tb* TKT protein. These peptides were designed to include the adjacent AA present in the TKTμ epitope, namely, THQPI, representing the antigenic determinants. These were: TKT1, GEDGPTHQPIEHLSA (15 AA); TKT2, DGPTHQPIEHLSALRAIPRLSV (22 AA); and TKT3, HDSIGLGEDGPTHQPIEHLS (20 AA) (Fig. 2B). Utilizing the OptimumAntigen design tool, we analyzed the potential antigenicity of the TKT peptides *in silico*. If the antigenicity is greater than 0.6 and the homology is less than 95%, then the peptide is considered to provoke a good immune response. The *in silico* analysis of the TKT peptides indicated that TKTμ, *M.tb* TKT1, and *M.tb*TKT3 showed a good antigenicity. The antigenicity of TKTμ was 0.96, and its homology was 35/58% (Mus musculus/Oryctolagus cuniculus). TKT1 was 1.72 with a homology of 39/46%, and TKT3 was 1.93 with a homology of 29/34% (Fig. S1). The *in silico* analysis indicated the lack of an antigenic property of the TKT2 peptide.

### Detection of specific IgG against *M.tb* TKT epitopes.

**FIG 3:**
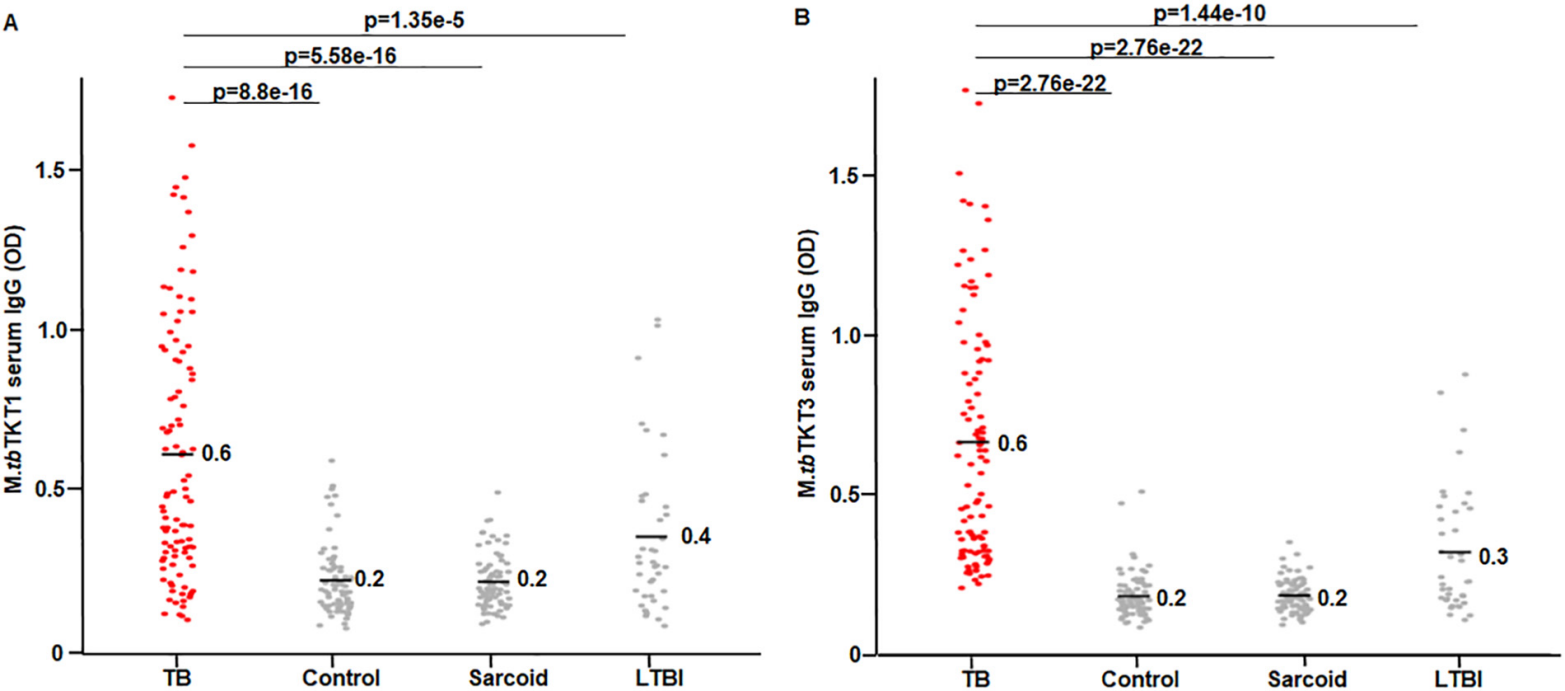
Evaluation of *M.tb* TKT1-specific IgGs and *M.tb* TKT3-specific IgGs in serum samples. The *M.tb* TKT specific IgGs and *M.tb* TKT3 specific IgGs in the sera of all study groups, namely, healthy subjects (*n* = 66), sarcoid (*n* = 76), smear positive TB subjects (*n* = 101), and LTBI (*n* = 49), measured via direct peptide ELISA, utilizing chemically synthesized *M.tb* TKT1 or *M.tb* TKT3 peptides. The comparison of the immunoreactivity of serum IgGs to *M.tb* TKT 1 and *M.tb* TKT 3 peptides is presented as OD values at 450 nm. (A) The OD values for the IgGs against *M.tb* TKT1 are shown as (Mean ± SD). TB sera (0.6 ± 0.39), healthy controls (0.2 ± 0.11), sarcoidosis (0.2 ± 0.08), and LTBI (0.4 ± 0.24). As shown, the TB sera display significantly higher ODs, compared to the controls (*P =* 8.8E−16), sarcoid (*P =* 5.58E−16), and LTBI (*P =* 1.35E−5). (B) OD for IgGs against *M.tb* TKT3, shown as (Mean ± SD). The TB sera (0.6 ± 0.35), healthy controls (0.2 ± 0.07), sarcoidosis (0.2 ± 0.05) and LTBI (0.3 ± 0.18). The TB sera exhibited significantly higher OD values of IgGs against *M.tb* TKT3, compared to the controls (adj. *P =* 2.76E−22), sarcoid (adj. *P =* 2.76E−22), and LTBI (adj. *P =* 1.44E−10).

We investigated whether the designed *M.tb* specific TKT epitopes (TKT1 and TKT3) have a better specificity and sensitivity to detect IgG in sera from active TB patients, compared to TKTμ. A similar algorithm was applied to develop and standardize the ELISA for each of the peptide sequences, as is described in Materials and Methods. The immunoreactivities to the *M.tb* TKT1 epitope are presented as OD values (Fig. 3A). As shown, the OD mean value in active TB was 0.61 ± 0.39, which was significantly different, compared to the OD values of the healthy controls (adj. *P* value = 8.8E−16), sarcoidosis (adj. *P* value = 5.8E−16), and LTBI (adj. *P* value = 1.35E−16). Similarly, we detected TKT3-specific IgGs in all of the study subjects via ELISA. The OD mean values against *M.tb* TKT3 in active TB was 0.62 ± 0.35, which was significantly different, compared to the OD values of the healthy controls (adj. *P* value = 2.76E−22), sarcoidosis (adj. *P* value = 2.76E−22), and LTBI (adj. *P* value = 1.44E−10) (Fig. 3B). These data indicate that both the *M.tb* TKT1 and TKT3 peptides bind to specific IgG in sera from active TB and that the sera of active TB subjects exhibit significantly higher OD values, compared to all of the other groups, even LTBI.

### Classifications and ROC curves of TKTμ, TKT1, and TKT3.

**FIG 4:**
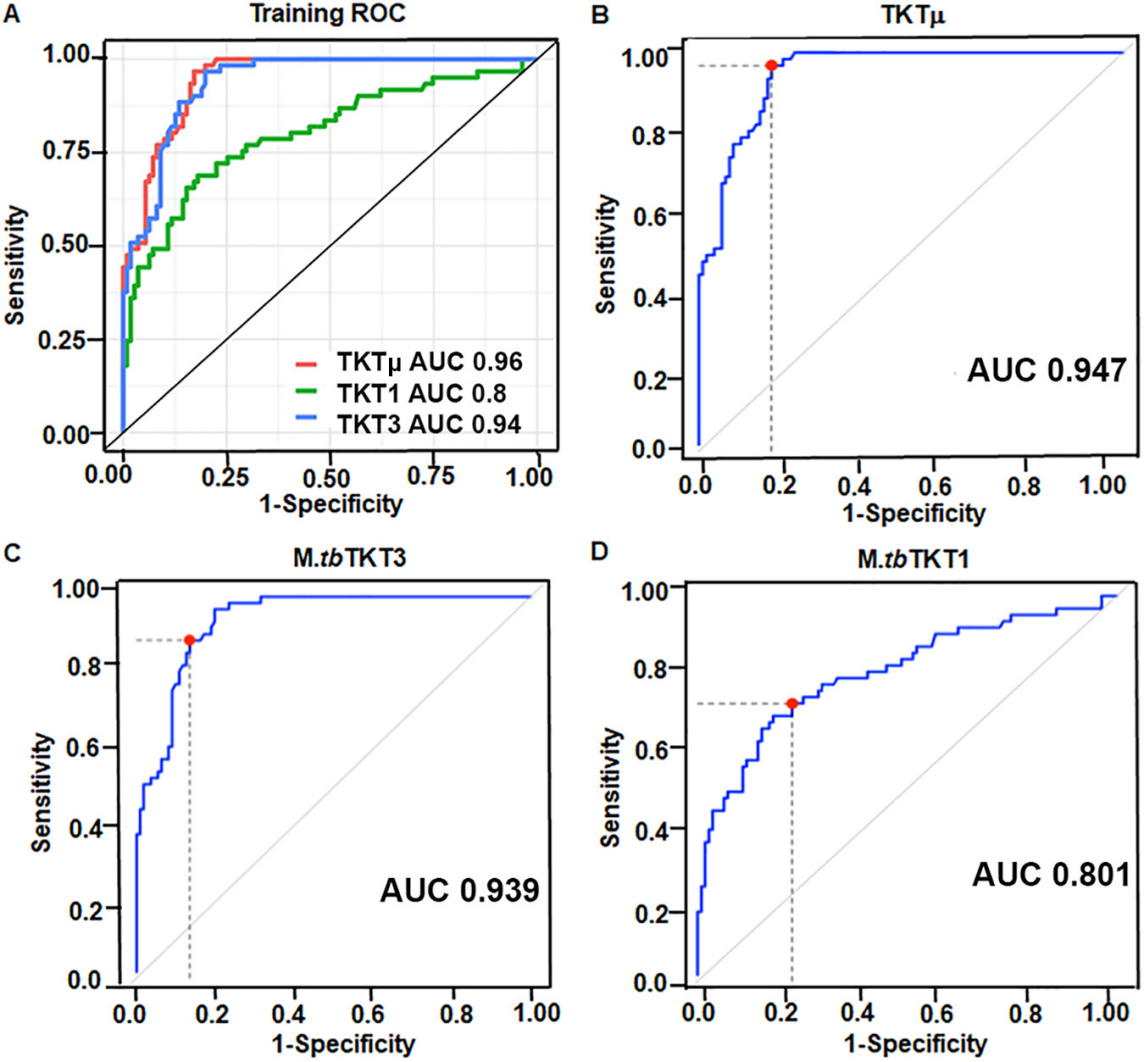
Classifications and receiver operating characteristic curves of TKTμ, TKT1, and TKT3. (A) Receiver operating characteristic (ROC) curves generated from the training set (60% of samples), using IgG specific OD values against TKTμ, *M.tb* TKT3, and *M.tb* TKT1. The training set was used to build a model and identify the best threshold for the IgG-specific epitopes. (B–D) The model from the training set was validated on the test set (40% of samples) by applying the thresholds in term of sensitivity, specificity, positive predictive value, negative predictive value, and accuracy. The panels show the performance of the TKTμ (panel B) *M.tb* TKT3 (panel C), and *M.tb* TKT1 (panel D) values on the test set, indicating the area under the curve (AUC) for each ROC. The red dot on each ROC curve (B–D) and its coordinates in the parentheses represent the optimal OD cutoff point, which is the point on the ROC curve closest to (0, 1) in term of 1-specificity and sensitivity. The OD value corresponding to this cut point is considered to be the threshold to determine a TB infected sample. That is, a sample is classified as a TB infection if its serum value is greater than or equal to its corresponding threshold value.

To develop a model to classify active TB from the other groups, we first randomly split the data of all subjects (*n* = 292) into the training and test sets at a ratio of 60/40. 60% percent of the samples were assigned to the training set, and the remaining samples were assigned to the test set (Table S2). Second, we generated ROC curves by using the *M.tb* TKT 1, *M.tb* TKT3, and TKTμ data from the training set (Fig. 4A). The *M.tb* TKT1 ROC curve yielded an area under the curve (AUC) of 0.8 (95% CI: 0.72 to 0.87) (Fig. 4A). *M.tb* TKT3 yielded an AUC of 0.95 (95% CI: 0.92 to 0.98). TKTμ yielded an AUC of 0.94 (95% CI: 0.91 to 0.97) (Fig. 4A). Based on the ROC curves, we identified the best OD thresholds for TKTμ, *M.tb* TKT1, and *M.tb* TKT3: 0.252, 0.293, and 0.267, respectively. Subsequently, we applied these OD thresholds to classify the samples in the test data set and to assess the sensitivity, specificity, positive predictive value [PPV], negative predictive value [NPV], and accuracy. The model from the training set was validated on the test set (40% of samples), and ROC curves were generated for all three peptides (Fig. 4B–D). A sample was considered “non-TB” if its serum IgG optical density was less than or equal to the corresponding threshold or “TB” otherwise. The classification with *M.tb* TKT3 on the test set yielded the highest sensitivity (0.845), and this was followed by TKTμ (0.817) and *M.tb* TKT1 (0.732). The specificities obtained by TKTμ, *M.tb* TKT3, and *M.tb* TKT1 on the test sets are 1, 0.95, and 0.875, respectively. The model using TKTμ again obtained a perfect PPV of 1, and this was followed by *M.tb* TKT3 with a PPV of 0.968 and *M.tb* TKT1 with a PPV of 0.912. The model with *M.tb* TKT3 had the highest NPV (0.776), and this was followed by TKTμ (0.755) and *M.tb* TKT1 (0.648), which were all measured on the test set. The accuracies using TKTμ and *M.tb* TKT3 were equally good (both 88.3%), whereas the accuracy using *M.tb* TKT1 was 78.4%. These results clearly show that the ELISA-based detection of IgG against both *M.tb* TKT3 and TKTμ has great sensitivity, specificity and accuracy to distinguish active TB subjects from healthy controls, sarcoidosis, and LTBI.

## DISCUSSION

**FIG 5:**
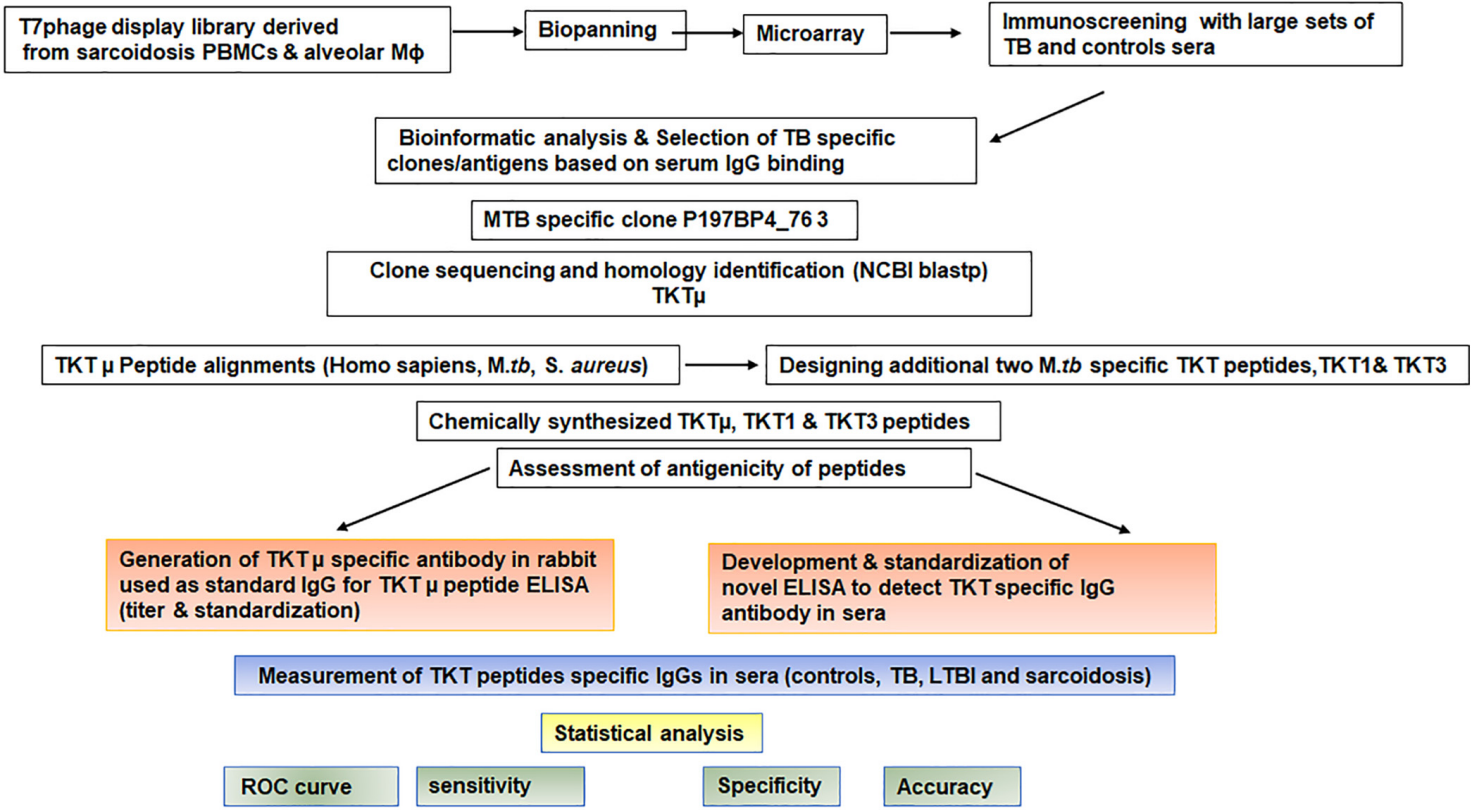
Schematic representation of discovery and development of the highly specific and sensitive peptide ELISA for detecting TKT-specific IgG antibodies in serum. Flow chart depicting the steps involved in the discovery of TB specific clones, utilizing the T7 phage display library and immune-screening, the identification of TKT epitopes, and the development of TKT peptide ELISA.

There is a need for a new conceptual approach with which to understand the complex host immune response to mycobacteria, including the mechanisms of humoral immunity (8, 23). The role of cell-mediated immunity in the defense against TB is well-known, but the role of antibodies in providing protection and their utilization as a diagnostic tool has not been well-established (9, 24). Most subjects developing TB are T cell competent, suggesting other possible immunological mechanism(s) underlying TB control (25). The elucidation of humoral immune responses to mycobacterial antigens seems to be difficult. Based on theoretical combinatorial calculations, the human antibody repertoire is estimated to include approximately 10^15^ members (26). This astronomical antibody repertoire is due to the somatic diversification and gene rearrangement of immunoglobulins, VDJ recombination, T cells and MHC class diversity and interaction, and, importantly, mutations in the *M.tb* genome, among others (27, 28). The evolutionary survival strategy of *M.tb* provided the advantage to balance species-specific host responses, thereby enabling the pathogen to remain dormant for a period of time by tuning down its metabolism, resisting host responses, and the actions of antibiotics (29–31). Recent studies highlight the importance of humoral immunity and B cells in the defense against *M.tb* (9, 32). Immunoglobulin promotes phago-lysosomal fusion and intracellular killing through opsonization and engagement with FcγR (33–36). Studies showed that antibodies present in the sera of LTBI subjects exhibit enhanced phagolysosomal maturation, inflammasome activation, and macrophage killing of intracellular *M.tb*, compared to the IgG present in the sera from the active TB subjects (37, 38). IgG antibodies may also gain access to the cytosol of *M.tb*-infected cells to promote the growth restriction of intracellular bacteria (16, 37, 38). The elucidation of humoral immune responses to mycobacterial antigens seems to be difficult. To this end, the detection of humoral immune responses can be achieved via peptide microarrays, which allow for the unbiased testing of several thousand unique epitopes that are displayed as linear peptides on a nitrocellulose-coated glass slide. Utilizing this approach, we developed a high-throughput method using a T7 phage display cDNA library that was derived from the BALs and whole leukocytes of sarcoidosis subjects, and we identified TKTμ as one of the top 10 classifier clones in distinguishing the sera of active TB patients from uninfected control sera with a high sensitivity and specificity (14, 15, 39, 40). Fig. 5 illustrates the schematic diagram of our approach toward the discovery and development of the highly specific and sensitive peptide ELISA for the serological detection of TKT-specific IgG antibodies. The TKT is a critical enzyme that catalyzes the cleavage of carbon-carbon bonds to transfer two ketol carbon units from donor ketose sugars, such as xylulose-5-phosphate, to acceptor aldose sugars, such as ribose-5-phosphate or erythrose-4-phosphate, thereby resulting in the production of sedoheptulose-7-phosphate or fructose-6-phosphate. TKT has a critical role in carbon branching in bacterial growth, including *M.tb* (17, 41), and the depletion of TKT changes the *M.tb* virulence (42). Because the dormancy/non-replicative phase of *M.tb* is characterized by a low metabolic rate and a lower *M.tb* cell division (29), we were enthusiastic to develop an ELISA for TKT-specific epitopes to test whether the IgG against any TKT epitope can differentiate between active TB and LTBI. As TKT plays a key role in the switch from dormancy to the proliferative phase, TKT specific IgG may uncover the differences between active TB and LTBI. Our study showed that both the TKTμ and *M.tb* TKT3 peptides are specific and sensitive to detect IgG against TKT that and both can be used to develop a sensitive direct peptide ELISA or POC test, such as a lateral flow, to differentiate TB from other conditions. In evaluating a large, diverse sample and developing a statistical classification model using training and test sets, the *M.tb* TKT3 epitope yielded the highest sensitivity (0.845), and it was followed by TKTμ (0.817) and *M.tb* TKT1 (0.732). The specificities obtained by TKTμ and *M.tb* TKT3 were 1 and 0.95, respectively. The specificity of the *M.tb* TKT1 peptide ELISA was the lowest (0.875). Despite a 75% sequence similarity between *M.tb* TKT1 and *M.tb* TKT3, there were differences in the sensitivity and specificity of these two peptides. These differences appear to be due to the five N-terminal AAs that may confer conformational changes that affect the affinity/avidity of IgG bindings to these epitopes. By applying the whole-genome sequencing of *M. tb* to clinical isolates, a recent study showed a significant genetic heterogeneity and a large number of single nucleotide variants (SNV) as well as that even one SNV can affect drug susceptibility and resistance (43). Interestingly, beside a well-described SNV in the rpoB gene leading Xpert MTB/RIF, they found numerous SNVs in metabolic genes, including the *M.tb* TKT gene (43, 44). Therefore, it is conceivable that the differences in the N-terminal AAs affect the antigenicity and the antibody responses.

Current, nonsputum-based POCs either detect various *M.tb* antigens, including lipoarabinomannan (LAM), culture filtrate proteins (CFPs), and ESAT-6 or detect host antibody responses to *M.tb* (5, 45). The sensitivities and specificities of the antigen-based POCs are lower than the ideal and are variable, based on the patient population, including hospitalized patients versus outpatients and HIV status (5, 46). Among these tests, the WHO endorsed a lateral flow urine (LAM) assay to aid in TB disease diagnoses in HIV-positive patients (46). LAM is a ubiquitous mycobacterial component that has a low specificity for distinguishing *M.tb* from nontuberculous mycobacteria (NTM), which is important in HIV-infected subjects (47). There are few serological tests available that quantitate the TB-specific antibodies in the serum. The InBios Active Tb Detect IgG ELISA, which uses a pool of *M.tb* antigens, was found to be sensitive in detecting active TB (48, 49). Other available tests for TB serodiagnosis utilize various components of *M.tb*, including the Ag60 complex (50, 51), the PPD (52), or a pool of membrane and secreted antigens from *M.tb* H37Rv (53). Most of these tests utilized either a single specific antigen or a pool of membrane or secreted *M.tb* antigens that contained various potential epitopes. Only a few studies included LTBI to assess specificity and sensitivity (54).

The target product profiles (TPPs) issued by the WHO that define nonsputum-based, rapid biomarker performance as a triage test recommend a sensitivity of >95% and a specificity of >80% to screen patients with suspected TB disease and to reduce the population that requires confirmatory tests (5, 8). Our data indicate that our TKT-specific ELISAs fulfill these criteria and are suitable for this purpose. Furthermore, the combination of three epitopes may increase the sensitivity and specificity of our test.

Efficacy studies from advanced TB vaccines that are designed to stimulate cell-mediated immunity failed to show consistent protection, warranting the need to harness other types of immunity, including antibody and B cell responses (8, 9, 37). One important consideration is whether TKTμ, *M.tb* TKT1, and *M.tb* TKT3 can serve as vaccine candidates. There are some important facts that may favor the potential utility of the TKT peptide as a vaccine candidate. First, our current data on TB subjects show the exhibition of antibodies against TKTμ, *M.tb* TKT3 and *M.tb* TKT1 epitopes. Second, we could evoke excellent antibody responses in TKTμ-immunized rabbits, suggesting a humoral response to the TKT epitopes. Third, the TKT enzyme is important for *M.tb* bacterial growth, and the IgG antibody levels were different in TB subjects versus LTBI subjects. Further experiments in animal models of *M.tb* infection can provide invaluable information.

Our study has certain noteworthy limitations. First, like any other human study, it is prone to bias due to unmeasured confounders. Second, prospective studies or the measurement of TKT antibodies in collected sera at the completion of antituberculosis treatments can provide insight into the value of treatment success. Third, further testing is required in subpopulations of immunocompromised subjects, such as HIV patients. Fourth, assessments of TKT antibodies in the sera of subjects that have positive cultures and are smear positive for NTM species are required to evaluate the diagnostic accuracy of this test. Because the IgG subclasses may affect the bacterial clearance as well as the Fcγ receptor activation, further studies are needed to evaluate the TKT-specific IgG subclasses.

## MATERIALS AND METHODS

### Chemicals and antibodies.

The ELISA plates were purchased from R&D Systems (Minneapolis, MN). The goat anti-human IgG HRP-linked antibody was purchased from Abcam (Cambridge, MA). The blocking buffer was purchased from Bio-Rad (Bio-Rad, Hercules, CA).

### Patient selection.

This study was approved by the institutional review boards (IRB) at Wayne State University and at the Detroit Medical Center. All of the methods were performed in accordance with the human investigation guidelines and regulations of the IRB (protocol no. 055208MP4E) at Wayne State University. All of the experiments were performed in accordance with the guidelines and regulations of the investigation of human subjects. Sera were collected from 4 groups: (i) healthy volunteers (*n* = 66), (ii) sarcoidosis subjects (*n* = 76), (iii) pulmonary TB patients (*n* = 101), and (iv) latent TB (*n* = 49). All study subjects signed to express their written informed consent. Sera from patients with active and latent tuberculosis were obtained from the Foundation for Innovative New Diagnostics (FIND, Geneva, Switzerland). All TB patients had smear positive sputum. The LTBI subjects had negative smear and culture but positive interferon gamma release assays (IRGs).

**Alignment of the TKT sequences from Homo sapiens, Mycobacterium tuberculosis (*M.tb*), and Staphylococcus aureus (*S. aureus*).** The amino acid sequences of TKT were obtained from UniProtKB: P9WG25 (TKT_MYCTU), A0A0B4J1R6 (TKT_HUMAN), and Q6G9L6 (TKT_STAAS). The sequence alignment and homology of TKT was done using the T-Coffee Multiple Sequence Alignment program. The FASTA files of the TKT amino acid (AA) sequences of three species were submitted to the T-Coffee alignment program and were illustrated in ClustalW format.

### TKT peptide ELISA.

The TKT peptide was chemically synthesized by Genscript (Piscataway, NJ). A MicroPlate (R&D Systems) was coated with TKT peptide at a concentration of 500 ng/mL (100 μL/well) overnight at room temperature (RT). The next day, the plates were washed 3× with washing buffer (PBS with 0.05% Tween 20). The plates were blocked at RT for 1 h with blocking buffer (Bio-Rad). After blocking, the plates were washed 3× with washing buffer, and individual serum samples (1:500 in blocking buffer) were added into wells in duplicate and incubated at RT for 2 h. After 2 h, the plates were washed 3× and then incubated with the goat anti-human IgG HRP-linked secondary antibody (Abcam, Waltham, MA) at RT for 90 min. The plates were washed 4×, and 100 μL/well TMB substrate mixture (R&D Systems) was added for 20 min at RT. The reaction was stopped via the addition of 50 μL of 2N H_2_SO_4_ to each well. The optical density (OD) was measured at 450 nm using a microplate reader (BioTek).

### Generation of the rabbit polyclonal antibody against the TKTμ peptide.

The rabbit polyclonal anti-TKTμ antibody was custom generated by Genscript (Piscataway, NJ). Briefly, the TKTμ peptide was conjugated to the carrier protein KLH, and 0.2 mg TKTμ peptide-KLH conjugate with Freud’s complete adjuvant was administered subcutaneously to each rabbit (New Zealand strain). After three booster doses of the conjugated TKTμ peptide (0.2 mg/rabbit) on days 14, 28, and 42, the sera from the immunized rabbits were collected on day 49 and pooled. The polyclonal anti-TKTμ IgGs were purified using the antigen affinity purification method.

### Statistical analyses.

The power and sample size were calculated using G*Power (55). The estimated effect size was 0.5 to 0.6 with a power of 90% and a false discovery rate (FDR) of 1 to 5%. The required sample sizes were *n* = 48 for the TB patients and *n* = 50 for the non-infected individuals (healthy controls). To reach a higher power near 100% and to be able to validate these results on an independent set, we increased the sample sizes and included sarcoidosis subjects (*n* = 76) and LTBI subjects (*n* = 49) as disease controls.

The analyses of the ELISA data were performed in the R programming language. We applied a two-tailed *t* test to determine whether the measurements of the IgG-specific TKT antibodies in the TB sera were significantly different, compared to those of the other groups. Subsequently, the significant scores (*P* values) were corrected for multiple comparisons using the FDR (56). To build a model for each IgG against the TKT-epitopes to distinguish the TB samples from the non-TB samples, we randomly split the data into training and test sets at a ratio of 60/40. The model is defined as the optimal cut point on the receiver operating characteristic (ROC) curve that was obtained from the training set closest to the point (1, 0). A sample was classified as a TB infection if its serum IgG ODs against specific TKT were greater than or equal to the OD threshold that corresponded to this cut point. We assessed the ability to correctly identify TB and non-TB samples in the test set of each model based on the sensitivity, specificity, positive predictive value, negative predictive value, and accuracy when applying the model to the test set.
