# Supplementary material for: Discovery of Novel Transketolase Epitopes and the Development of IgG-Based Tuberculosis Serodiagnostics
Source: Microbiol Spectr. 2023 Jan 18;11(1):e03377-22. doi: 10.1128/spectrum.03377-22 (PMC9927582; doi:10.1128/spectrum.03377-22)
Supplement: Supplemental file 1 — Supplemental material. Download spectrum.03377-22-s0001.pdf, PDF file, 0.1 MB [file spectrum.03377-22-s0001.pdf]

Discovery of Novel Transketolase Epitopes and the Development of IgG-based Tuberculosis Serodiagnostics

Jaya Talreja[$1], Changya Peng [$1], Tuan-Minh Nguyen[2], Sorin Draghici[2] and Lobelia Samavati [1, 3*]

[1]Department of Medicine, Division of Pulmonary, Critical Care and Sleep Medicine, Wayne State University School of Medicine and Detroit Medical Center, Detroit, MI 48201

[2] Department of Computer Science, Wayne State University; Detroit; MI 48202, USA

[3]Center for Molecular Medicine and Genetics, Wayne State University School of Medicine, Detroit, MI 48201

[$] Authors contributed equally.

Table of contents

Supplementary Table S1	Page 2
Supplementary Figure S1	Page 3
Supplementary Table S2	Page 4

Supplementary Table S1: TKT-μ peptide ELISA using different dilutions of custom-made rabbit anti-TKT-μ IgG.

**Antibody Dilution**	**1:1,000**	**1:2,000**	**1:4,000**	**1:8,000**	**1:16,000**	**1:32,000**	**1:64,000**	**1:128,00**	**1:256,00**	**1:512,000**	**Blank**
**Anti-TKT-μ OD (450nm)**	2.609	2.579	2.527	2.451	2.294	2.033	1.781	1.412	1.007	0.683	0.055
**Control IgG OD (450nm)**	0.170	0.118	0.083	0.072	0.063	0.059	0.055	0.053	0.059	0.054	0.057

Supplementary Table 1: TKT-μ peptide ELISA using different dilutions of custom-made rabbit anti-TKTμ IgG. TKT-μ peptide ELISA was done via standardized TKT peptide ELISA. Two-fold serial dilutions of custom made anti-TKT-μ antibody (1:1000-1:512,000) in duplicates were used. Rabbit IgG (1:1000-1:512,000) was used as a control. The ELISA plate was read for OD value at 450nm using plate reader.

Supplementary Figure S1. Antigenic determinants plot of *M.tb* TKT.

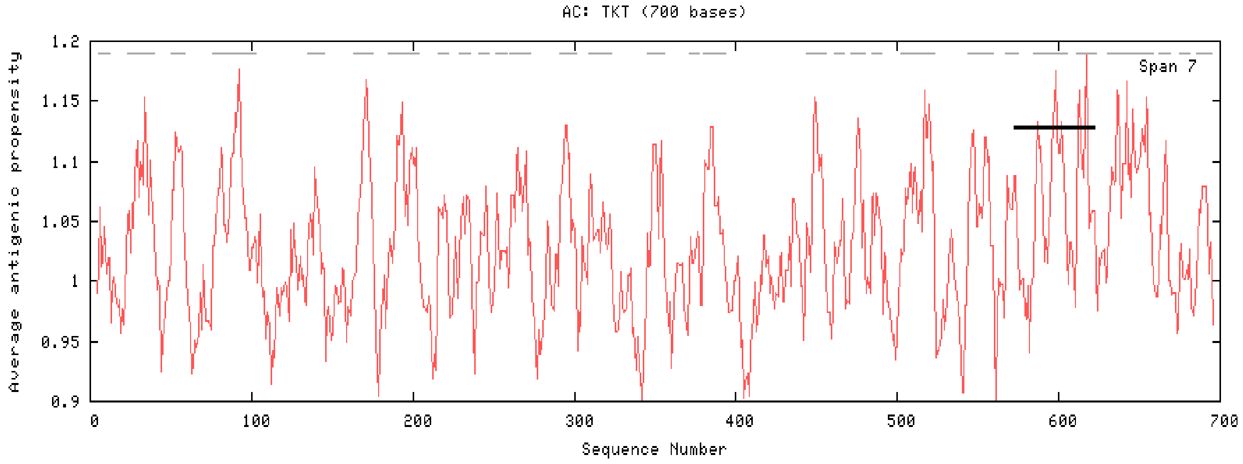

Supplementary Figure 1. Antigenic determinants plot of *M.tb* TKT. Antigenic peptides of *M.tb* TKT were determined using the method of Kolaskar and Tongaonkar, an antigenic peptide prediction tool provided by Immunomedicine group. There were 30 antigenic determinants in *M.tb* TKT sequence. TKT-µ, *M.tb* TKT1 and *M.tb* TKT3 peptides represent the antigenic determinants in the region spanning between sequence number 500-600.

Supplementary Table S2: Sample allocation of subjects in different groups for training and test sets.

**Supplementary Table S2. Sample allocation in training and testing sets**		
	Training	Testing
TB subjects	61	40
Controls	41	25
Sarcoidosis	46	30
LTBI	29	20

Supplementary Table S2: Sample allocation of subjects in different groups for training and test sets. To develop a model to classify active TB from other groups, subjects were randomly split into the training and test sets with a ratio of 60/40.
